# Unraveling socioeconomic determinants of health-related behavior, reception of information, and perceptions on disease disclosure at the time of the COVID-19 pandemic: did health insurance curb the disparities in the Philippines?

**DOI:** 10.1186/s12889-024-18264-9

**Published:** 2024-03-12

**Authors:** Josue Antonio G. Estrada

**Affiliations:** Independent Researcher, Manila, Philippines

**Keywords:** Socioeconomic inequalities, COVID-19, Health insurance, Public health emergency, Health behavior, Stigma, Access to information, Developing countries

## Abstract

**Background:**

The study uncovers micro and macro socioeconomic disparities in terms of health behavior, disease perception, and reception of information. Furthermore, findings shed light on the possible role of health insurance on access to information, disease perception and the adoption of preventive behaviors in the context of a public health emergency such as the COVID-19 pandemic.

**Methods:**

This study employed a cross-sectional design using the Philippine Demographic and Health Survey (DHS). With a total of 29,809 respondents, it evaluated the individual or household and systemwide socioeconomic determinants of four different outcomes: receipt of information, disease perception, uptake of free preventive services, and treatment-seeking behavior. In addition to logistic regression models with the socioeconomic variables as the independent variables, models for the evaluation of the moderating effect of insurance ownership were fitted. Predicted probabilities were reported for the analysis of moderating effects.

**Results:**

Findings show that individual and householdsocioeconomic determinants affected health-behavior and access to or receipt of information pertinent to the COVID-19 pandemic. Both education and wealth affected the receipt of information such that individuals in more advantaged socioeconomic positions were at least 30% more likely to have received information on COVID-19. Wealth was also associated to treatment-seeking behavior. Regional differences were seen across all dependent variables. Moreover, the study provides evidence that ownership of insurance can close education-based gaps in the uptake of free vaccination and COVID-19 testing.

**Conclusion:**

It is imperative that targeted efforts be maximized by utilizing existing strategies and mechanisms to reach the marginalized and disadvantaged segments of the population. Health insurance may give off added benefits that increase proficiency in navigating through the healthcare system. Further research may focus on examining pathways by which health insurance or social policies may be used to leverage responses to public health or environmental emergencies.

**Supplementary Information:**

The online version contains supplementary material available at 10.1186/s12889-024-18264-9.

## Introduction

More than 2 years have passed since the severe acute respiratory syndrome coronavirus 2 or SARS-CoV-2 virus first emerged and rapidly spread all over the globe, causing a pandemic that has profoundly impacted the lives of billions of people. As of mid-2023, there is an estimated 6.9 million deaths caused by the coronavirus (COVID-19) disease and although the World Health Organization (WHO) has declared the end of the public health emergency, many remain affected by productivity losses, disability, and other persistent health and social effects of the pandemic [1–3]. To make things worse, the mid- and long-term effects on socioeconomic factors and quality of life at both the micro and macro levels are expected to cause a continuing scourge in the years to come [4, 5].

The literature documents inequalities not just in infection and mortality but also in other aspects and effects of the COVID-19 pandemic among and within countries [5–8]. On a larger scale, low- and middle-income countries have taken the brunt of the ill effects of this pandemic while within countries, socioeconomically disadvantaged populations were forced to take several steps further away from achieving a good quality of life [9–12]. Differences in contextual factors such as socioeconomic status give rise to disparities in terms of access to information, healthcare, and social services and even in health-related behavior and disease-associated stigma which subsequently affected infection, mortality rates and the emergence of long-term economic and social effects [13–18].

The Philippines, a lower-middle income country, was one of the most severely affected countries in the Western Pacific and Southeast Asian regions [19–21]. As of July 2023, almost 67 thousand COVID-19 related deaths have occurred in the country– the second highest number of deaths in Southeast Asia [1]. Despite vast improvements in health service quality and coverage and achievement of several important milestones such as the implementation of landmark legislature for universal health care and excise tax for tobacco and alcohol which increased resources for health, the country’s healthcare system remains lacking as problems in resource allocation and inadequate capacity remain [22, 23]. At the beginning of the pandemic, the country only had 8 physicians per 100,000 population and 2,335 critical care beds [24, 25]. Health capacity and resources are unevenly distributed with much of these concentrated in urban and highly-developed areas while the rural population, comprising almost half of the total population, rely mostly on primary care centers and community and some tertiary hospitals with limitations on laboratory and diagnostic testing capacity, health personnel, equipment and supplies and as a consequence, possess very limited surge capacity [26–28]. Governance is decentralized hence, while national strategies are in place, local governments are mainly responsible for the crafting of plans and service provision [27]. Localities create disaster risk reduction and management plans but these are crafted mostly for environmental emergencies and natural disasters and do not usually cover plans for the mitigation of the effect of epidemics and other public health emergencies [29]. At the onset of the pandemic, strategies such as “community quarantines”, internal movement and travel restrictions, and mass testing were implemented but certain challenges were encountered. For instance, due to limited infrastructure, testing was implemented mostly in urban areas. Additionally, as Filipinos spend more than 10 hours a day in the internet, there was an added challenge on risk communication with the emergence of various sources of misinformation [26, 30, 31]. In the hopes of bringing attention and building more equitable healthcare systems, the study examined the socioeconomic determinants of health and associated disparities in terms of receipt of COVID-19 related information, health behavior at the time of the pandemic, and perceived stigma. This analysis based on the Philippine Health and Demographic Survey (DHS) and done among adults aged 18 and above provide an understanding of the different aspects of the country’s response to the COVID-19 pandemic. Furthermore, this study explored and offered insights into the potential role that the national health insurance program (NHIP) under the Philippine Health Insurance Corporation (PHIC), popularly called Philhealth, and other health insurance, play in the curbing of socioeconomic disparities in COVID-19 related behavior, refusal to disclose disease, and access to information. Classical economic theories posit that the possession of health insurance may disincentivize individuals to take preventive efforts to remain healthy. This is termed ex-ante moral hazard and is suggested to stem from an informational asymmetry, where the insurer cannot observed some of the actions of the insured [32, 33]. However, the fact that health insurance covers only part of the expenses may mean that the extent of moral hazard in terms of health behavior may not be significant enough in most cases [34]. In fact, most empirical evidence has shown, that health insurance can, to some degree, induce health-related behavior change in a beneficial manner [34–37]. The social cognitive theory asserts that individuals require information about health risks and uses these information to develop self-regulatory and risk-reduction skills however self-efficacy, is a key factor to adoption of these skills [38]. Theoretically, insurance ownership may also impart health consciousness and health-related information such as those on risk reduction leading to behavior change and adoption of appropriate health-related behavior. Furthermore, ownership of health insurance may be considered a resource that can impart skills essential for efficient navigation of the healthcare system [39]. In the Philippines, the introduction of reforms towards universal healthcare has, in theory, made health insurance available to everyone through several schemes such as the point-of-care enrolment [23]. However, information asymmetry and poor health literacy resulting from poor communication may hinder individuals from gaining knowledge of their entitlement to health insurance and to financial and nonfinancial benefits that come with it [40]. Therefore, this study measures if entitlement to benefits of health insurance in the Philippine context modifies the relationship between socioeconomic determinants and COVID-19 related behavior and access to information. More than providing a picture of disparities that emerged during the COVID-19 pandemic, results of this study could contribute to the knowledge base of the indirect role of interventions such as health insurance in curbing health inequalities or specifically modifying preventive behaviors.

## Methods

### Study design, sample and data

The study used cross-sectional data from the 2022 Philippine DHS, a nationwide survey conducted from May 2 to June 22, 2022 and included a more than 30,000 households. The Philippine DHS 2022 has a response rate of 99.2% [41]. Included in this study are survey respondents aged 18 and above who are *de jure* or usual residents of the interviewed households. Three sets of analytic samples were considered were used for the study– an overall analytic sample, an analytic sample for the outcome “uptake of free preventive services”, and an analytic sample for the outcome “Treatment-seeking behavior” which was derived for those who confirmed having experienced COVID-19 related symptoms since January 2020. Figure S1 in the appendix shows how these analytic samples were derived. Data used was extracted from the household survey module. A complete case analysis was done, hence, observations with missing information for relevant variables were excluded. A multistage stratified sampling design was used for the survey, details of which are discussed in the DHS report [41].

The study used publicly available de-identified data which were requested from the DHS program. The survey has undergone ethical review under the institutional board of ICF International.

### Measures

#### Outcome variables

The study considered a total of four outcome variables, each representing a dimension of health behavior and access to information. These variables are listed in Table 1.

**Table 1:** COVID-19 related outcomes and their definition and categorization

Outcome	Definition
Receipt of information about COVID-19	Those who stated that they have received health information about the COVID-19 pandemic through television, print or digital sources were coded as 1
Refusal to disclose disease	Those who responded yes or don’t know to the question, “If a member of your family got infected with COVID-19, would you want it to remain a secret?” were coded as 1
Uptake of free preventive services	Based on the question, “Which of these health programs initiated by your local government unit (provincial, city/municpal, or barangay) did you or any of your household members avail in the past 30 days?”. Those who availed “free COVID-19 vaccination” or “free COVID-19 RT PCR testing”in the past 30 days were coded 1
Treatment-seeking behavior	Among respondents who’ve had COVID-19 or experienced related symptoms since January 2020, those who sought treatment or medical attention were coded as 1

Health-seeking behavior was represented by 2 variables: (a) uptake of free vaccination and testing services and (b) seeking consultation and treatment due to COVID-19 or COVID-19 related symptoms. For the latter, the study considered only those who answered “Yes” to the question: “Since January 2020, have you ever had COVID-19 or COVID-19 symptoms such as fever or chills, cough, shortness of breath or difficulty breathing, fatigue, muscle or body aches, headache, loss of taste or smell, sore throat, congestion or runny nose, nausea or vomiting, or diarrhea?”. These variables, to a certain degree, measure risk perception and preventive behavior which affect disease transmission and effectiveness of social measures. In a study by Ye and Lyu [42], risk perception mediated the relationship between public trust and COVID-19 infection. Another outcome variable considered was the respondent’s decision to make COVID-19 diagnosis of a family member a secretor refusal to disclose disease may represent perceptions of stigmatization and has been shown to be an aspect of the overall stigma experience of an individual [43, 44]. Refusal to disclose disease may be related to courtesy stigma or stima-in-association which refers to the perceived and experienced stigma of associates from the general public toward themselves [45]. A related concept, affiliate stigma, on the other hand, is the internalization of stigma among associates and describes the extent of self-stigmatization and cognitive, behavioral and affective responses of the associates [46, 47]. Lastly, access to information, a crucial factor affecting adoption of preventive and healthy behavior, was represented by perceived access to information, which in turn, was assessed through the respondent’s receipt of health-related information about COVID-19 [48, 49].

### Socioeconomic determinants

There were three socioeconomic determinants considered in the analysis. At the micro level, differences in levels of wealth and education were assessed. Respondents were assessed as either poor (belonging to the poorest 40% of the population) or nonpoor based on the household wealth index of the Philippine DHS. Wealth index scores, a composite measure of a household’s cumulative living standard, were estimated using principal component analysis (PCA) from information on a household’s ownership of selected assets. The estimation of these scores are described further in relevant documents [50, 51]. For education, highest educational attainment was used and classified as either “with secondary education or higher” or “lower than secondary education”. For the macro level determinant, the study used regions classified in quartiles according to the reported poverty incidence in 2021 [52]. The quartiles were classified according to regionwide statistics, with the first quartile containing the regions with the highest poverty incidence.

### Health insurance ownership

This study considers knowledge on ownership of insurance which was defined in 3 categories: (1) no health insurance, (2) entitled to PhilHealth and (3) entitled to Philhealth and other health insurance. Those who reported entitlement to PhilHealth are either members or dependents of listed members.

Philhealth, being the national health insurance, is mandatory for employees in public and private institutions while voluntary for the workers in the informal economy. Sponsored members or those whose premiums are subsidized by the government mostly include indigent citizens. As mentioned, the advent of universal health care law has, in theory, made PhilHealth available to everyone, however, as evidenced by results of the DHS, a sizeable proportion of the population is still unaware of their entitlement to the social health insurance [41].

Due to sparse data issues, respondents were classified into the three aforementioned groups based on their responses when asked of their ownership of health insurance. Less than 1% of the overall analytic sample declared being entitled to benefits of other insurance only. However, with the assumption that Philhealth is considered as the main source of health insurance, while health insurance from other sources such as that from the private market is often considered supplementary, the small proportion of respondents who responded having other insurance only were classified as having both Philhealth and other insurance. The potential impact of the adoption of this categorical approach was minimal as illustrated in the appendix (see Appendix 2).

### Confounders

A review of literature reveals possible factors which may confound the relationship of socioeconomic factors to COVID-19 response related outcomes [53–58]. Among these, the following variables were considered in the analysis: sex (male or female), age (categorized as < 20, 21–30,31–40, 41–50, 51–60, and 60 and above), area of residence (urban or rural), household size (number of de jure household members categorized as 1–3, 4–6, 7 and above), relationship to household head (categorized as self, spouse, child, or others), awareness of programs on COVID-19 (defined as awareness of free vaccination and COVID-19 testing and classified as aware or unaware), belief that COVID-19 can be prevented (defined as one’s belief that COVID-19 can be prevented through vaccination or other social measures and classified as believing or does not believe) and use of internet (defined as use of internet for health-related reasons and classified as yes or no).

### Statistical analysis

All analyses were weighted based on the complex survey design of the data in use. Descriptive statistics were calculated for all participants and analyses were conducted using R (version 4.3.1). The threshold for statistical significance was set at an alpha of 0.05.

Binary logistic regression was used to conduct two levels of analysis. The first level included models wherein the main effects of the socioeconomic determinants were determined, and insurance ownership was considered a confounder, together with the other identified variables. Adjusted odds ratios (aOR) and their respective 95% confidence intervals were reported for each socioeconomic determinant. For the second level of analysis, the moderating effect of insurance ownership was assessed through the introduction of interaction terms between each socioeconomic variable and health insurance ownership. The Wald test was used to determine if there were significant differences in the relationship of the socioeconomic determinants with the COVID-19 related outcomes across levels of insurance ownership. Relationships were further elaborated using predictive margins. To provide further illustration, predicted probabilities were plotted.

## Results

### Descriptive statistics

The total analytic sample is comprised of 29,809 individuals which comprises 98% of the total household sample interviewed in the 2022 Philippine DHS. For assessments involving the outcome, “uptake of free preventive services”, the study included 27,999 respondents while for “treatment-seeking behavior”, the study included 5,130 respondents who was diagnosed with COVID-19 or exhibited symptoms of the disease. Table 2 shows the descriptive statistics of the overall sample in terms of weighted proportions.

**Table 2:** Descriptive statistics on the overall analytic sample using weighted proportions (*n* = 29,809)

Variable	Insurance ownershipn, n (%wt)						Total n (%wt)	
	Unsure/ No health insurance (*n* = 7,169)		With Philhealth only (*n* = 21,238)		With Philhealth and other insurance (*n* = 1,402)			
**Wealth**								
Poor	3,899	(53.9)	7,818	(36.8)	562	(23)	11,953	(40)
Nonpoor	3,334	(46.1)	13,427	(63.2)	842	(77)	17,929	(60)
**Educational attainment**								
Below secondary	2,235	(30.9)	5,396	(25.4)	362	(10.6)	7,710	(25.8)
At least secondary	4,998	(69.1)	15,849	(74.6)	1,042	(89.4)	22,172	(74.2)
**Region** ^**a**^								
I	1,259	(17.4)	2,422	(11.4)	180	(11)	3,825	(12.8)
II	1,714	(23.7)	4,993	(23.5)	334	(27.8)	7,112	(23.8)
III	1,548	(21.4)	4,823	(22.7)	309	(16.2)	6,574	(22)
IV	2,712	(37.5)	9,008	(42.4)	581	(45.1)	12,371	(41.4)
**Sex**								
Male	1,844	(25.5)	5,248	(24.7)	352	(29.2)	7,500	(25.1)
Female	5,389	(74.5)	15,997	(75.3)	1,052	(70.8)	22,382	(74.9)
**Age in years**								
< 20	386	(5.3)	387	(1.8)	37	(1.5)	786	(2.6)
21 to 30	1,468	(20.3)	2,422	(11.4)	191	(13.5)	4,064	(13.6)
31 to 40	1,461	(20.2)	4,525	(21.3)	300	(27.4)	6,395	(21.4)
41 to 50	1,504	(20.8)	4,610	(21.7)	306	(27.3)	6,514	(21.8)
51 to 60	1,584	(21.9)	3,930	(18.5)	270	(17.5)	5,737	(19.2)
61 and above	825	(11.4)	5,375	(25.3)	299	(12.9)	6,365	(21.3)
**Area of residence**								
Urban	3,660	(50.6)	11,430	(53.8)	751	(61.4)	15,987	(53.5)
Rural	3,573	(49.4)	9,815	(46.2)	653	(38.6)	13,895	(46.5)
**Household size**								
1 to 3	2,966	(41)	8,519	(40.1)	566	(39.8)	12,042	(40.3)
4 to 6	3,392	(46.9)	9,879	(46.5)	656	(48.7)	13,955	(46.7)
7 and above	875	(12.1)	2,847	(13.4)	183	(11.6)	3,885	(13)
**Relationship to household head**								
Self	2,799	(38.7)	9,475	(44.6)	607	(43.9)	12,909	(43.2)
Spouse	3,197	(44.2)	9,581	(45.1)	630	(45)	13,417	(44.9)
Child	817	(11.3)	1,506	(7.1)	114	(8.3)	2,435	(8.2)
Others	415	(5.7)	680	(3.2)	53	(2.8)	1,130	(3.8)
**Awareness of COVID-19 programs** ^**b**^								
unaware	1,938	(26.8)	5,502	(25.9)	369	(29)	7,859	(26.3)
aware	5,295	(73.2)	15,743	(74.1)	1,035	(71)	22,023	(73.7)
**Belief in prevention of COVID-19** ^**c**^								
No	574	(7.9)	1,500	(7.1)	101	(5.6)	2,149	(7.2)
Yes	6,662	(92.1)	19,737	(92.9)	1,303	(94.4)	27,730	(92.8)
**Use of internet for health reasons** ^**d**^								
No	5,446	(75.3)	14,085	(66.3)	951	(52.8)	20,230	(67.7)
Yes	1,787	(24.7)	7,160	(33.7)	453	(47.2)	9,652	(32.3)

^a^classification based on poverty incidence with regions in the first quartile having the highest level of weighted regional poverty incidence in 2021

^b^ defined as awareness of free vaccination and COVID-19 testing and classified as aware or unaware

^c^ defined as one’s belief that COVID-19 can be prevented through vaccination or other social measures

^d^ defined as use of internet for health-related reasons

Among those with both PhilHealth and other health insurance, 77% are nonpoor while 89.4% finished at least secondary education. Regions with the least poverty incidence are most represented in the survey with more than 40% of respondents belonging to these regions. Most of the respondents are female, aged 31 and above, living in urban areas, and in households with 4 to 6 members. Respondents are also generally shown to be aware of free vaccination and testing services, and an overwhelming majority are aware of and believe in measures to prevent COVID-19. Lastly, most individuals use the internet for health reasons. This was observed regardless of ownership of insurance.

**Fig. 1:**
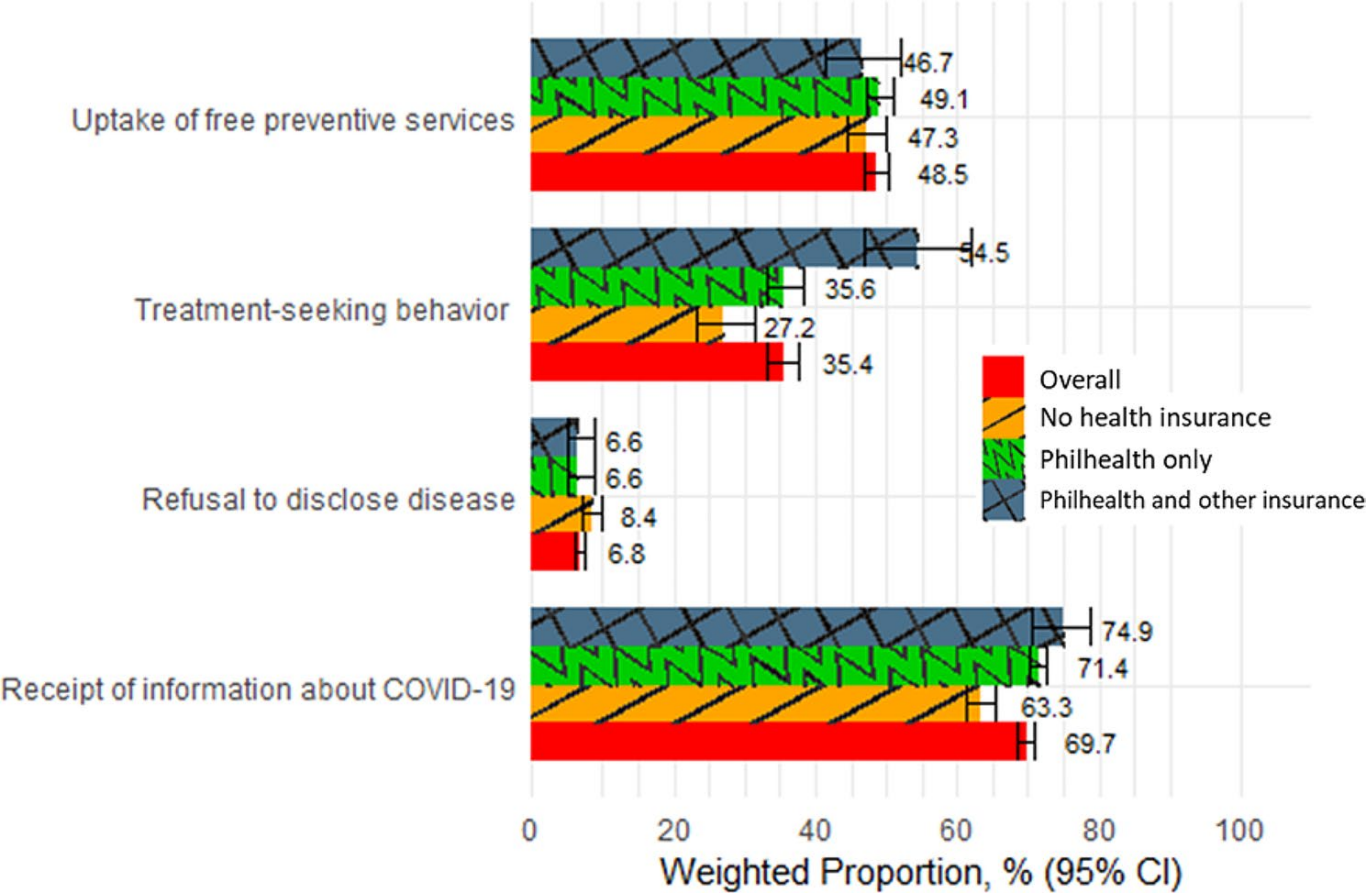
Weighted proportion and corresponding 95% confidence intervals of the respondents with the outcome, by category of health insurance ownership

Figure 1 illustrates the weighted proportion and 95% confidence intervals of the outcomes in the analytic samples. As shown in the figure, approximately 47–49% of respondents availed free preventive services while less than 55% of those who had COVID-19 or related symptoms sought treatment or consultation. Meanwhile, less than 10% of respondents agreed or were uncertain about keeping a diagnosis of COVID-19 in the family a secret. Considering that this is a public health emergency, penetration of information among the respondents can be considered inadequate, with only approximately 70% of respondents reporting receipt of information on the pandemic. Generally, those with Philhealth and other insurance had more favorable outcomes.

### Socioeconomic disparities

The first level of analysis revealed that individual socioeconomic determinants affected health-behavior and perceived access to or receipt of information pertinent to the COVID-19 pandemic. Both education and wealth affected the receipt of information such that individuals in more advantaged socioeconomic positions were 30% more likely to have received information on COVID-19. Education and wealth-based disparities were also seen in the uptake of COVID-19 vaccination and free testing services while for treatment-seeking behavior, only wealth was found to be significantly associated. However, if we look at results for the macro-level socioeconomic determinant, regional disparities can be seen across all outcomes considered. Furthermore, regions with the lowest poverty incidence were generally found to have more beneficial outcomes. Table 3 presents the results of the multivariate models.

**Table 3:** Adjusted Odds Ratios (aOR) of each study outcome and socioeconomic determinants

Socioeconomic factor	Outcomes aOR (95% Confidence Interval)			
	Receipt of information about COVID-19	Refusal to disclose disease in the family	Uptake of free preventive services	Treatment-seeking behavior
**Wealth**				
Poor	ref	ref	ref	ref
Nonpoor	1.30 (1.18,1.44)*	0.98 (0.82,1.16)	1.15 (1.02,1.3)*	1.74 (1.4,2.18)*
**Educational attainment**				
Below secondary	ref	ref	ref	ref
At least secondary	1.34 (1.23,1.47)*	0.90 (0.72,1.12)	1.13 (1.004,1.27)*	1.18 (0.92,1.5)
**Region**				
I	ref	ref	ref	ref
II	1.35 (1.15,1.6)*	0.5 (0.34,0.75)*	1.01 (0.79,1.31)	0.99 (0.77,1.28)
III	0.93 (0.79,1.09)	0.43 (0.28,0.67)*	0.94 (0.7,1.25)	1.22 (0.94,1.59)
IV	1.23 (1.03,1.48)*	0.51 (0.35,0.74)*	1.68 (1.25,2.24)*	1.74 (1.29,2.34)*

*Abbreviation* aOR- adjusted odds ratio; CI- confidence interval

^a^classification based on poverty incidence with regions in the first quartile having the highest level of weighted regional poverty incidence in 2021

### Insurance ownership as a moderator

**Fig. 2:**
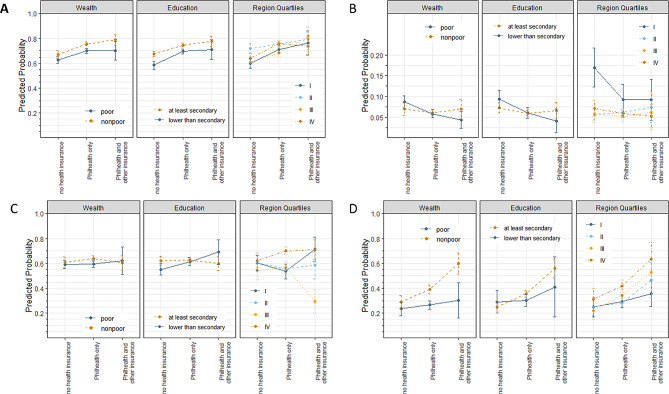
Predicted probabilities of socioeconomic determinant across categories of self-reported health insurance ownership for (**A**) receipt of information about the COVID-19 pandemic, (**B**) refusal to disclose disease, (**C**) uptake of free preventive services, and (**D**) treatment-seeking behavior

Figure 2 shows the plotted predicted probabilities for each socioeconomic determinant across categories of health insurance ownership in each COVID-19 related outcome. Shown in Table 4 are the predicted probabilities of each level of socioeconomic determinant for each study outcome, stratified by the different categories of health insurance ownership. Testing for interaction between the socioeconomic determinants and health insurance ownership revealed that health insurance modified the relationship between education and uptake of free preventive services, specifically vaccination and free COVID-19 testing. Among those without health insurance, the probability of availing preventive services was significantly higher in those with at least secondary education. Ownership of health insurance– either PhilHealth alone or in combination with other health insurance– curbed the disparity between the two levels of educational disposition. Among people with at least Philhealth as insurance, living in regions with the lowest poverty incidence gives significantly higher probabilities of getting vaccinated and availing testing services than living in regions with the highest levels of poverty. When considering people without health insurance, one can see significant differences in the levels of refusal to disclose disease, with “poorer” regions experiencing higher levels of refusal to disclose COVID-19 diagnosis in the family. For populations who own insurance, no regional differences were seen with regards to this outcome. On the other hand, a notable result can be seen in the case of household wealth. Among those who reported that they don’t own any health insurance, poorer individuals have shown a higher probability to perceive disclosure of disease negatively but among those who reported ownership of insurance, individuals from nonpoor households were more likely to keep diagnosis of COVID-19 in the family a secret. Differences on predicted probabilities were, however, not significant. Marginally significant results can also be seen with wider socioeconomic disparities among insured populations in terms of (a) education and refusal to disclose disease, and (b) wealth and treatment-seeking behavior.

**Table 4:** Predicted probabilities (%) for each outcome at certain levels of each socioeconomic determinant and stratified by ownership of insurance

Variable interactions		Receipt of information about COVID-19			Refusal to disclose disease			Uptake of free preventive services			Treatment-seeking behavior		
		Predicted probability,%	Difference, pp	p-value	Predicted probability,%	Difference, pp	p-value	Predicted probability,%	Difference, pp	p-value	Predicted probability,%	Difference, pp	p-value
**Wealth**													
No health insurance	poor	62.6 (60.1,65.1)*	ref	0.3075	8.7 (7.2,10.2)*	ref	0.0351	59.2 (55.9,62.5)*	ref	0.3775	23.4 (17.7,29.1)*	ref	0.0895
	nonpoor	66.9 (63.7,70)*	4.2 (0.3,8.1)*		6.9 (5.4,8.4)*	-1.7 (-3.6,0.1)		61 (56.6,65.4)*	1.8 (-2.6,6.2)		28.8 (23.6,34.1)*	5.6 (-2,13.2)	
with Philhealth only	poor	69.9 (68,71.8)*	ref		5.8 (4.8,6.8)*	ref		59.7 (56.8,62.6)*	ref		26.6 (23.1,30)*	ref	
	nonpoor	75.5 (73.9,77.1)*	5.6 (3.3,7.9)*		6.1 (5.4,6.7)*	0.3 (-0.9,1.4)		63.8 (61.6,66.1)*	4.2 (1,7.4)*		39.2 (35.7,42.7)*	12.6 (7.2,18.1)*	
with Philhealth and other insurance	poor	70.2 (62.8,77.6)*	ref		4.3 (2.2,6.4)*	ref		62 (50.9,73.1)*	ref		30.2 (16.2,44.3)*	ref	
	nonpoor	79 (74.8,83.1)*	9.4 (0.7,18.1)*		6.9 (4.6,9.3)*	2.5 (-0.6,5.6)		60.4 (54.5,66.2)*	-1.7 (-14,10.6)		59.7 (51.3,68.1)*	29.2 (13.2,45.2)*	
**Education**													
No health insurance	lower than secondary	58.6 (55.3,61.8)*	ref	0.3667	9.4 (7.3,11.5)*	ref	0.0585	55.2 (50.5,59.9)*	ref	0.0113	29.1 (20,38.3)*	ref	0.2165
	At least secondary	67.3 (65.1,69.6)*	8.6 (5.2,12)*		7.2 (6,8.4)*	-2.1 (-4.2,0.1)		62.2 (58.8,65.6)*	6.9 (2.2,11.6)*		25 (20.2,29.8)*	-4.4 (-15.4,6.7)	
with Philhealth only	lower than secondary	69.5 (67.5,71.6)*	ref		6.1 (4.8,7.4)*	ref		61.1 (58,64.3)*	ref		30.4 (25.6,35.1)*	ref	
	At least secondary	74.7 (73.3,76.2)*	5.2 (3.1,7.4)*		5.9 (5.3,6.5)*	-0.2 (-1.6,1.3)		62.8 (60.6,64.9)*	1.6 (-1.6,4.9)		35.7 (33,38.5)*	5.3 (-0.3,11)	
with Philhealth and other insurance	lower than secondary	71 (63,79)*	ref		4.1 (1.2,6.9)*	ref		69.1 (59.1,79.2)*	ref		41.1 (17,65.2)*	ref	
	At least secondary	77.6 (73.8,81.5)*	7.1 (-1.2,15.3)		6.5 (4.5,8.5)*	2.4 (-0.7,5.5)		59.9 (54.3,65.5)*	-9.5 (-20.3,1.4)		56 (48.2,63.7)*	14.7 (-9.8,39.3)	
**Region in quartiles**													
No health insurance	I	59.9 (55.8,64)*	ref	0.0068	16.9 (12.2,21.7)*	ref	0.0012	60.3 (54,66.6)*	ref	> 0.0001	24.7 (16.8,32.7)*	ref	0.268
	II	71.8 (67.8,75.8)*	11.6 (6.3,17)*		5.6 (4.2,6.9)*	-11.2 (-15.9,-6.5)*		60.9 (55.6,66.2)*	0.5 (-7.4,8.5)		25.3 (18.2,32.4)*	0.6 (-10.6,11.8)	
	III	60.8 (56.3,65.3)*	0.9 (-4.9,6.7)		5.8 (3.8,7.8)*	-11 (-16,-6)*		54.2 (47.2,61.2)*	-6.1 (-15.4,3.3)		21.9 (14.7,29)*	-3 (-14.3,8.2)	
	IV	64.1 (60.4,67.8)*	4.1 (-1.5,9.8)		7.1 (5.3,9)*	-9.7 (-14.5,-4.9)*		62.3 (56.3,68.3)*	1.9 (-7,10.8)		31 (21.8,40.1)*	6.5 (-6.3,19.2)	
with Philhealth only	I	71.1 (68.3,74)*	ref		9.2 (5.3,13)*	ref		53.7 (47.7,59.7)*	ref		29.6 (24.5,34.6)*	ref	
	II	75.4 (73.1,77.7)*	4.3 (1,7.6)*		6.1 (5.2,7)*	-3.1 (-7.1,1)		55.5 (52.1,58.8)*	1.8 (-4.8,8.4)		27.9 (24.1,31.8)*	-1.6 (-7.9,4.6)	
	III	68.2 (65.6,70.7)*	-3 (-6.6,0.7)		5.1 (4,6.1)*	-4.1 (-8.2,0)*		56.7 (52,61.5)*	3.1 (-4.2,10.4)		34.4 (29.6,39.1)*	4.8 (-2,11.5)	
	IV	75.9 (73.4,78.4)*	4.7 (0.8,8.7)*		5.8 (5,6.7)*	-3.3 (-7.1,0.4)		70.2 (67.1,73.3)*	16.6 (9.6,23.6)*		41.7 (36.8,46.7)*	12.1 (4.7,19.5)*	
with Philhealth and other insurance	I	76.3 (66.4,86.2)*	ref		9.2 (4.1,14.2)*	ref		71.3 (61.2,81.4)*	ref		35.6 (25.2,45.9)*	ref	
	II	79.3 (73.4,85.2)*	3.3 (-9.2,15.7)		7.3 (3.6,11)*	-1.8 (-7.8,4.2)		58.4 (47.6,69.2)*	-13.2 (-28.2,1.8)		46.6 (32.3,60.8)*	17 (1.4,32.7)*	
	III	81.4 (74.1,88.7)*	5.6 (-7.9,19)		6.2 (1.9,10.5)*	-2.9 (-9.3,3.6)		29.4 (20.1,38.7)*	-41.9 (-55.4,-28.3)*		53 (40.7,65.2)*	27.9 (10.9,44.8)*	
	IV	73.5 (67.1,79.9)*	-3.1 (-15.8,9.7)		5.2 (2.4,8.1)*	-3.8 (-9.4,1.8)		71 (63,79)*	-0.3 (-13.8,13.2)		63.6 (50.5,76.7)*	10.7 (-6.2,27.6)	

Abbreviation pp- percentage points

^a^p-value for interaction

## Discussion

Results of the study show that socioeconomic disparities at the individual and regional levels were observed in perceived access to information, perceptions, response, and behaviors at the time of the COVID-19 pandemic. At an individual or household level, wealth and education were seen to be related to receipt of health information regarding COVID-19 and uptake of free preventive services. At the regional level, poverty incidence, which may be indicative of governance efficiency and availability and equitability of social services, has been found to give rise to inequalities in health behavior, receipt of information and refusal to disclose disease. These study’s findings are important considering that healthcare navigation and individual responses, including disease perceptions shape, not only the individual’s outcome, but on a collective level, the population’s health outcomes [59–61]. In the context of the COVID-19 pandemic, these disparities in preventive behavior and disease perception may have implications in dynamics based on disease transmission, severity of outcomes and mid- and long-term health and economic effects. On the other hand, access to information especially in times of public health emergencies is essential to mitigate the health and social impacts of the crisis [62]. Acknowledging the disparities in health-related behavior and access to information is an important component of a targeted, comprehensive, and effective response to public health emergencies such as the COVID-19 pandemic.

In terms of perceived access to information and uptake of free preventive services, both wealth and education were considered significant determinants with those in higher socioeconomic positions gaining health advantage more than their poor and less educated counterparts. Health literacy is generally expected to be higher in more educated individuals as they have greater perceived access to information and are more able to interpret and assess the verity of information [63–65]. In the same way, wealth endows greater amounts of financial, informational and social resources. Furthermore, people in higher socioeconomic status are more likely to develop skills that can enable them to maximize use of available resources and adopt beneficial health behaviors that can lead to health advantage. Among individuals who were confirmed to have COVID-19 or showed related symptoms, disparities were primarily driven by differences in levels of wealth such that individuals from nonpoor households were 74% more likely to seek consultation. Disparities in wealth can be partlyexplained by the fact that the cost of healthcare or possible out-of-pocket expenditure may outweigh perceived risk. It may also be because patient isolation may entail opportunity costs due to loss of livelihood and considerations about the precarity in the quality of care or conditions and experience of being isolated. While Philhealth provides reimbursements, these may be inadequate when considering factors such as increased cost of high quality or satisfactory care– in the context of a public health emergency involving a highly transmissible infectious agent - and indirect costs associated with treatment-seeking such as waiting time, transportation costs, use of technology with higher transactional costs, and uncertainty of seeing a physician [66, 67]. In general, out-of-pocket (OOP) payments made by PhilHealth members are still significant as, on average, PhilHealth is only able to cover 56% of healthcare costs with limited coverage for outpatient services [68, 69]. In inpatient settings, mild to moderate pneumonia due to COVID-19 can lead to OOP payments ranging from USD 538 to USD 925– a huge amount considering that the median monthly basic pay of time-rated workers on full-time basis across all industries in 2020 was only pegged at approximately USD 272 [70, 71]. Another possible explanation is that poor insurance literacy especially on complex administrative processes for claims may deter individuals from seeking care [72]. Furthermore, nonpoor individuals may have enough resources to purchase higher quality care in healthcare facilities with safer environments thus the observed disparity [73]. This phenomenon is demonstrated in significant wealth-based disparities which persisted regardless of ownership of insurance.

Results for the macro-level socioeconomic determinant, on the other hand, show that there were regional disparities in the health-related behavior, refusal to disclose disease and receipt of information. People in regions with the highest poverty incidences were clearly disadvantaged across all study outcomes. Receipt of information regarding the pandemic and treatment-seeking behavior were generally better in richer regions. While there is a maldistribution of resources among regions in the Philippines, those considered more developed and highly urbanized have more resources for healthcare in general, and have more efficient local governance, which translates to more available infrastructure, human resource for health and healthcare services and more spending on social protection programs [27, 74]. Efficiency in turn, increases trust, ultimately leading to higher uptake of free services [75, 76].

This analysis also suggests a possible unraveling of the role of health insurance on socioeconomic disparities. Knowledge on entitlement to insurance was associated with better outcomes and nonsignificant socioeconomic disparities in some of the outcomes. A study by Soni [77] also provides evidence that health insurance ownership may help improve health-related behavior especially among people in low socioeconomic positions. For micro-level socioeconomic status, ownership of insurance has shown to curb the education-based disparities in uptake of free vaccination and testing. In the case of free service utilization such as vaccination, information costs are higher than financial costs as trust is a major prerequisite for uptake [78]. This result may demonstrate health insurance’s ability to bridge the education-based gap in uptake of preventive services. In this case, possession of insurance may have imparted additional health knowledge or increased health literacy thus increasing one’s propensity to avail free services regardless of level of education [63]. Ownership of health insurance may have led to increased access to and interaction with health care providers which may improve knowledge and establish trust with the healthcare system thereby increasing the probability of availing preventive services [79]. The same mechanisms may have also operated when considering differences in the negative perception on disease disclosure across different regions. As seen in the results, higher poverty incidence on a regional level was associated with higher probability for a certain degree of stigmatization of COVID-19. Negative perceptions on disease disclosure or the decision to keep diagnosis of COVID-19 a secret may be indicative of of self-perception of stigma and can arise from low health literacy. Possession of health insurance may increase access to factual information about the disease, hence buffering the effect of differential transmittal of information across regions [80]. Theoretical effects of increased health literacy that comes with having health insurance extend to regional disparities in the uptake of free vaccination. Probabilities of vaccination and free testing were generally higher among those with health insurance. However, a clear difference in uptake of services was seen between the regions with the highest poverty incidence which means that the “richest” regions may have complemented the effects of health insurance with efficiency in the relay of information. It is also important to note that regions with higher incidences of poverty have bigger rural populations, less infrastructure, and less number of health facilities and providers [27, 74].

Notably, in some instances, wider socioeconomic disparities were seen in groups insured with Philhealth only and Philhealth and other insurance. In the case of treatment-seeking behavior, the availability of insurance packages for COVID-19 may encourage individuals to seek care [66, 67, 71]. However, since most facilities are inundated by the volume of patients, accessibility to a facility able to accommodate the individual may require greater financial costs in terms of transportation costs or even time costs [81]. Furthermore, access to quality and safe healthcare services such as telemedicine or the ones provided in high-level healthcare facilities, comes with significant costs as well. Individuals from poor households might have limited capacity and access to resources for this expense. Of note, however,are negative relationships between knowledge of entitlement to health insurance and socioeconomic disparities in the outcome, “refusal to disclose disease”. Considering education or wealth, socioeconomic disparities were seen to be wider among insured populations and probabilities of keeping COVID-19 diagnosis of a family member a secret were higher among socioeconomically advantaged populations. Filipinos are culturally family-centered and families in the Philippine context, are usually extended and have closeknit ties, often living together in one roof [82]. Several psychological responses may lead people who are at a higher risk of contagion to perceive courtesy stigma and internalize it [83]. In a study by Duan et al. [83], high level of education was associated with perception of courtesy and affiliate stigma. They further explained that well-educated individuals may be more sensitive to the behavior of others and perceive these as discrimination [84]. In the case of both wealth and education, previous studies indicate that people with higher socioeconomic status have a higher likelihood for mental health problems such as anxiety or depression thus making them more vulnerable to perceive and internalize stigma [85–87]. There is a dearth in literature on the effect of health insurance on perceptions and experiences of stigmatization in disease. Kagaigai and Greperrud [88] in a study involving voluntary health insurance, however, found a strong relationship between risk aversion and insurance enrolment. This might have aggravated the already existing perceptions of stigma experienced by people in higher socioeconomic status. In the case of this analysis, risk aversion may be related to the risk of psychosocial and mental costs of stigmatization associated with having a family member diagnosed or isolated in a facility [89, 90].

Results of the second level of analysis suggest that, in terms of financial risk protection, Philhealth alone offers inadequate support to close wealth-based gaps in health behavior and access to information. However, beyond this, results may provide a peak into the role health insurance may play in levelling the playing field in terms of access to information and engaging in favorable health behavior. Findings may provide evidence that having a certain level of health insurance literacy may either be a valuable resource in healthcare navigation or an indicator of having health-related behaviors and skills essential in maximizing resource utilization to gain health advantage. In general, results of this study add to the literature on the importance of addressing socioeconomic inequalities to address health problems especially in times of public health emergencies. It also offers insights on the role of health insurance in closing these gaps. There is however, a need to investigate the dynamics of insurance and stigmatization. Further research can focus on the pathways by which different types of health insurance or social policies may be used to leverage responses to epidemics and other natural disasters.

Some limitations should be considered. First, threats to accuracy and measurement exist as data may be subject to errors in recall and social desirability bias. The survey, being done in a way that ensures confidentiality of responses, may alleviate desirability bias concerns. Second, nonresponse to the outcome “uptake of free preventive services” may potentially have resulted to selection bias due to imbalances in the characteristics of responding and nonresponding individuals. Resulting effect estimates and interpretations should be taken with a grain of salt. Third, residual confounding is present due to the exclusion of some important variables. These variables may include incidence or prevalence of COVID-19 in the localities and information on other lifestyle factors or health-related behavior. Fourth, provincial differences may be a more significant determinant of macro-level socioeconomic status as “within-region” differences may still vary greatly, nevertheless, weighted regional estimates may still determine area-specific differences to some extent. Fifth, household wealth may seem to be a less accurate measure of one’s ability-to-pay however, studies have shown that the DHS wealth index is similar to other indicators of socioeconomic status and is adequate in the assessment of “absolute economic status” [51, 91]. Lastly, due to the cross-sectional nature of the study, associations found in the study do not assume causality.

## Conclusion

Three years after the pandemic emerged, countries have started to loosen restrictions, but the world’s experience has given important lessons in building resilience against public health emergencies. This paper shows that individual responses differ by socioeconomic determinant, with socioeconomically disadvantaged population exhibiting less favorable health behavior and experiencing less access to information pertaining to the pandemic. Furthermore, results show empirical evidence of a possible modification of health insurance on the effect of socioeconomic determinants on these outcomes. This may provide proof that health insurance can grant added benefit maybe in the form of increased health literacy or endowment of attributes that increase proficiency in navigating through the healthcare system. In light of the results, the study highlights that preparedness and responsiveness of the healthcare system are key components of an effective response to public health crises and emergencies. It is, therefore, imperative that targeted efforts be maximized by utilizing existing strategies and mechanisms to reach the marginalized and disadvantaged segments of the population. Mitigation of the immediate and long-term effects of such crises requires looking into contextual factors that might affect the equitable distribution of resources and response to healthcare and social needs.

### Electronic supplementary material

Below is the link to the electronic supplementary material.

Supplementary Material 1

## Data Availability

The dataset supporting the conclusions of this article is available in the DHS repository, www.dhsprogram.com.

## Abbreviations

COVID-19: Coronavirus disease 2019
CI: Confidence interval
DHS: Demographic and Health Survey
OOP: Out-of-pocket
PP: Percentage points
SARS-CoV-2: Severe Acute Respiratory Syndrome Coronavirus 2
