# Supplementary material for: Unraveling socioeconomic determinants of health-related behavior, reception of information, and perceptions on disease disclosure at the time of the COVID-19 pandemic: did health insurance curb the disparities in the Philippines?
Source: BMC Public Health. 2024 Mar 12;24:767. doi: 10.1186/s12889-024-18264-9 (PMC10935915; doi:10.1186/s12889-024-18264-9)
Supplement: Supplementary file 1 — Supplementary Material 1 [file 12889_2024_18264_MOESM1_ESM.docx]

Supplemental materials

**Appendix 1: Derivation of analytic samples**

**
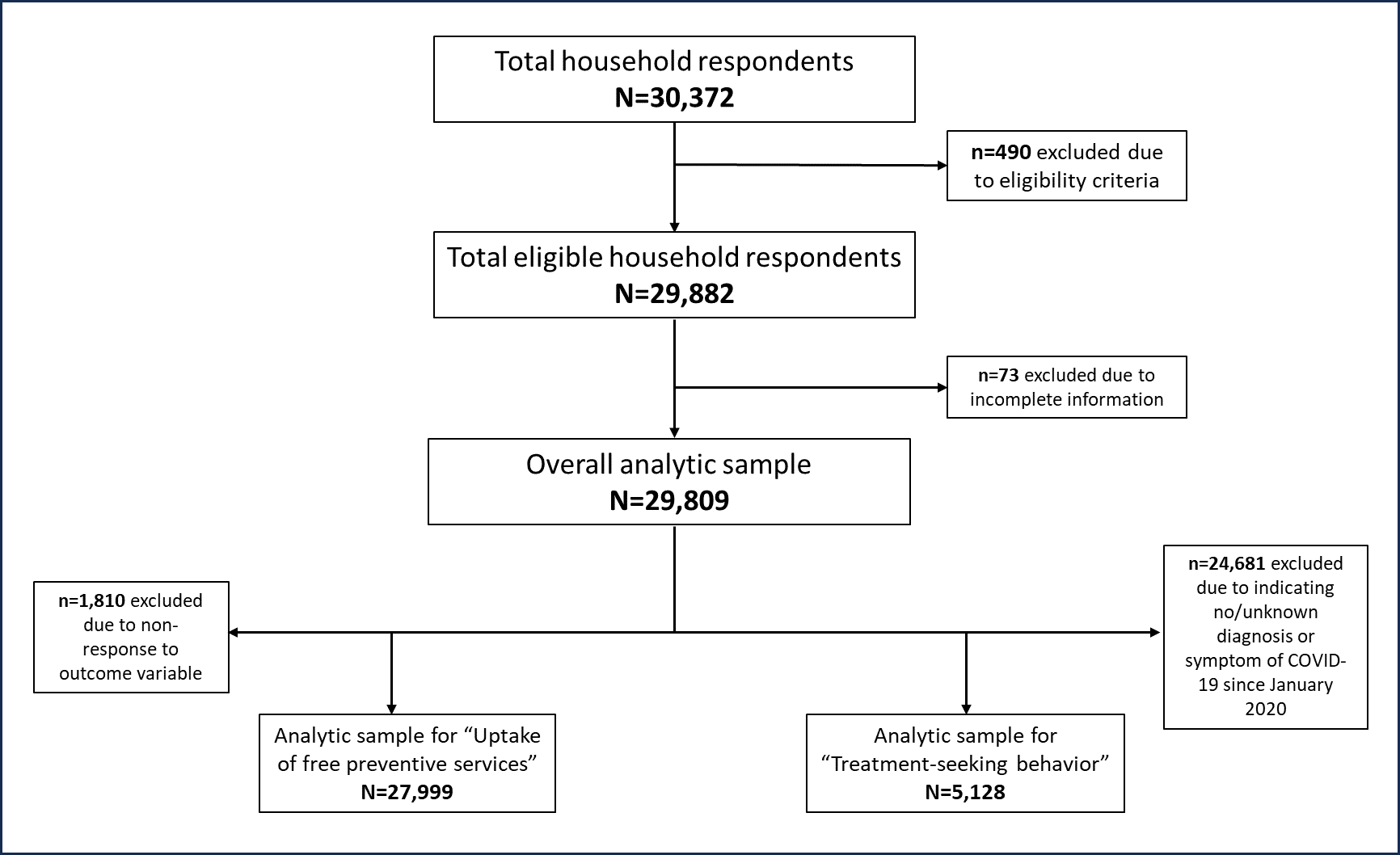
**

**Figure S1. Flowchart depicting formation of analytic samples from the total household respondents**

**Appendix 2: Results of sensitivity analysis**

The variable, health insurance ownership was comprised of four categories (Approach A): (a) “no insurance”, (b) “entitled to Philhealth only”, (c) “entitled to Philhealth and other insurance”, and (d) “with other insurance only”. Two forms of other insurance (i.e. Government Service Insurance System and Social Security System) were automatically categorized into PhilHealth since possession of either means that the respondent is enrolled either as contributor to PhilHealth. This leaves those who declared non-ownership of Philhealth but ownership of other health insurance such as those from HMOs and private insurance providers. The 4^th^ category includes only 66 observations. Considering that PhilHealth is the main health insurance provider, a new approach was adopted. In the new categorical approach (Approach B), those under category (d) was merged with category (c) with the assumption that they are enrolled members or dependents of Philhealth. For each of two outcomes: “Receipt of information about COVID-19” (Table S1) and “Refusal to disclose disease” (Table S2), logistic regression models were fitted, each with a different categorical approach of insurance status. Both models in each outcome were adjusted for the identified confounders. Wald tests were performed to test for differences in the coefficients. Change in odds ratios after adoption of the new categorical approach, were also quantified.

Sensitivity analyses conducted for both outcomes showed that there were generally no significant differences in estimates from both categorical approaches. Furthermore, after fitting the model for approach B, changes in the odds ratios from the first model involving approach A were minimal and were pegged at less than 5%. This justifies the use of categorical approach B in the main analysis. Tables S1 and S2 present the adjusted odds ratios for health insurance and the socioeconomic and demographic determinants of interest.

**Table S1. Summary of results for the regression models with the different categorical approaches with “receipt of information about COVID-19” as the outcome**

| **Variable** | **Categorical approaches** | | **% change in estimate** | **p-value** |
| --- | --- | --- | --- | --- |
|  | **Approach A** | **Approach B** |  |  |
| Health insurance ownership |  |  |  |  |
| no health insurance | ref | ref | ref | --- |
| with Philhealth only | 1.4 (1.2,1.5)* | 1.4 (1.2,1.5)* | 0 | 0.351 |
| with philhealth and other insurance | 1.4 (1.1,1.8)* | 1.4 (1.1,1.7)* | 2.7 | 0.1901 |
| with other insurance only | 0.7 (0.3,1.7) | --- | --- | --- |
| Wealth |  |  |  |  |
| poor | ref | ref | ref | --- |
| nonpoor | 1.3 (1.2,1.4)* | 1.3 (1.2,1.4)* | -0.1 | 0.2096 |
| Educational attainment |  |  |  |  |
| lower than secondary | ref | ref | ref | --- |
| secondary or higher | 1.3 (1.2,1.5)* | 1.3 (1.2,1.5)* | 0.1 | 0.3255 |
| Region |  |  |  |  |
| I | ref | ref | ref | ---- |
| II | 1.4 (1.1,1.6)* | 1.4 (1.1,1.6)* | 0 | 0.6722 |
| III | 0.9 (0.8,1.1) | 0.9 (0.8,1.1) | 0.1 | 0.2958 |
| IV | 1.2 (1,1.5)* | 1.2 (1,1.5)* | 0 | 0.8477 |
| Constant | 0.3 (0.2,0.4)* | 0.3 (0.2,0.4)* | 0.3 | 0.4804 |

**Table S2. Summary of results for the regression models with the different categorical approaches with “refusal to disclose disease” as the outcome**

| **Variable** | **Categorical approaches** | | **% change in estimate** | **p-value** |
| --- | --- | --- | --- | --- |
|  | **Approach A** | **Approach B** |  |  |
| Health insurance ownership |  |  |  |  |
| no health insurance | ref | ref | ref | --- |
| with Philhealth only | 0.8 (0.7,0.9)* | 0.8 (0.7,0.9)* | 0 | 0.3263 |
| with philhealth and other insurance | 0.8 (0.6,1.1) | 0.8 (0.6,1.1) | 1.7 | 0.2292 |
| with other insurance only | 0.4 (0.1,1.5) | --- | --- | --- |
| Wealth |  |  |  |  |
| poor | ref | ref | ref | --- |
| nonpoor | 1 (0.8,1.2) | 1 (0.8,1.2) | 0 | 0.3684 |
| Educational attainment |  |  |  |  |
| lower than secondary | ref | ref | ref | --- |
| secondary or higher | 0.9 (0.7,1.1) | 0.9 (0.7,1.1) | 0 | 0.7998 |
| Region |  |  |  |  |
| I | ref | ref | ref | ---- |
| II | 0.5 (0.3,0.8)* | 0.5 (0.3,0.8)* | 0 | 0.9728 |
| III | 0.4 (0.3,0.7)* | 0.4 (0.3,0.7)* | 0.1 | 0.3734 |
| IV | 0.5 (0.3,0.7)* | 0.5 (0.3,0.7)* | 0 | 0.8433 |
| Constant | 0.3 (0.2,0.5)* | 0.3 (0.2,0.5)* | 0.3 | 0.4073 |

**Appendix 3: Analysis of missing values and potential selection bias**

As detailed in Figure S1, the analytic sample for “uptake of free preventive services” excluded individuals without a response on the outcome. Though only 6% of the overall analytic data were excluded, there is a possibility of testing for the nature of “missingness” in the outcome. To test for the possibility of selection bias, presented in the Table S3 are the proportion of missing responses taking into account the survey design weights, and measured by socioeconomic and demographic characteristics. Chi-square statistics were used to present crude associations of the selected factors with missingness. As shown in Table S3, factors such as sex educational attainment, age and area of residence are crudely associated to “missingness” hence the assumption of the data being missing not at random may be taken. Implications of these findings are discussed in the paper.

**Table S3. Descriptive statistics for missing values of the outcome “Uptake of free preventive services”**

| **Variable** | **Total observations** | **Weighted proportion, %** | | **p-value** |
| --- | --- | --- | --- | --- |
|  |  | **Nonmissing** | **Missing** |  |
| Wealth |  |  |  |  |
| poor | 15,081 | 92.9 | 7.1 | 0.9739 |
| nonpoor | 14,801 | 92.9 | 7.1 |  |
| Educational attainment |  |  |  |  |
| below secondary | 8,842 | 91.6 | 8.4 | 0.0025 |
| at least secondary | 21,040 | 93.4 | 6.6 |  |
| Region quartile |  |  |  |  |
| I | 6,357 | 94.9 | 5.1 | 0.0524 |
| II | 8,228 | 92.1 | 7.9 |  |
| III | 6,662 | 92.6 | 7.5 |  |
| IV | 8,635 | 93 | 7.1 |  |
| Sex |  |  |  |  |
| Male | 7,433 | 90.5 | 9.5 | <0.0001 |
| Female | 22,449 | 93.7 | 6.3 |  |
| Age, in years |  |  |  |  |
| <20 | 804 | 91 | 9 | 0.0377 |
| 21 to 30 | 3,927 | 92.3 | 7.7 |  |
| 31 to 40 | 6,223 | 93.9 | 6.1 |  |
| 41 to 50 | 6,537 | 92.9 | 7.1 |  |
| 51 to 60 | 5,824 | 93.6 | 6.4 |  |
| 61 and above | 6,567 | 92 | 8 |  |
| Area of residence |  |  |  |  |
| Urban | 11,391 | 91.9 | 8.1 | 0.0011 |
| Rural | 18,491 | 94 | 6 |  |
